# Alteration of mitochondrial function in arthropods during arboviruses infection: a review of the literature

**DOI:** 10.3389/fphys.2025.1507059

**Published:** 2025-02-13

**Authors:** María E. Santana-Román, Santos Ramírez-Carreto, Paola Maycotte, Victoria Pando-Robles

**Affiliations:** ^1^ Centro de Investigaciones Sobre Enfermedades Infecciosas, Instituto Nacional de Salud Pública, Cuernavaca, Morelos, Mexico; ^2^ Centro de Investigación Biomédica de Oriente (CIBIOR), Instituto Mexicano del Seguro Social (IMSS), Puebla, Mexico

**Keywords:** mitochondria, arthropods, infection, arbovirus, metabolite

## Abstract

Arthropods serve as vectors for numerous arboviruses responsible for diseases worldwide. Despite their medical, veterinary, and economic significance, the interaction between arboviruses and arthropods remains poorly understood. Mitochondria in arthropods play a crucial role by supplying energy for cell survival and viral replication. Some arboviruses can replicate within arthropod vectors without harming the host. Successful transmission depends on efficient viral replication in the vector’s tissues, ultimately reaching the salivary glands for transmission to a vertebrate host, including humans, via blood-feeding. This review summarizes current knowledge of mitochondrial function in arthropods during arbovirus infection, highlighting gaps compared to studies in mammals and other pathogens relevant to arthropods. It emphasizes mitochondrial processes in insects that require further investigation to uncover the mechanisms underlying arthropod-borne transmission.

## Introduction

Mitochondria are cytoplasmic organelles that perform various vital cellular functions, including the regulation of cell death, control of intracellular calcium levels, lipid homeostasis, metabolic signaling, immune regulation, and the generation of energy (Abate et al., 2020; Spinelli and Haigis, 2018). Approximately 90% of the energy that cells acquire is produced in the mitochondria in the form of adenosine triphosphate (ATP), mainly through electron transport chain (ETC) coupled to oxidative phosphorylation (OXPHOS) (Mills et al., 2017; Supinski et al., 2020).

Mitochondria consists of an outer membrane (OMM) and an inner membrane (IMM) separated by the intermembrane space. The IMM forms invaginations known as cristae, surrounding the mitochondrial matrix (Chapman et al., 2020; Joubert and Puff, 2021; Roger et al., 2017). The OMM is only slightly selective for cytosolic solutes through porins such as voltage-dependent anion channel (VDAC), which mediate interactions between the mitochondria and the rest of the cell. In contrast, IMM is highly selective for solutes and houses five protein complexes (Complexes I-V) that constitute the ETC for OXPHOS. OXPHOS begins with the extraction of electrons from reduced nicotinamide adenine dinucleotide (NADH) in Complex I (CI) or flavin adenine dinucleotide (FADH₂) in Complex II (CII). These electrons are sequentially transported to Complex III (CIII) via coenzyme Q₁₀ and to Complex IV (CIV) by cytochrome c. In Complex IV, electrons react with molecular oxygen (O₂) to produce water (H₂O). During electron transport, protons (H⁺) are pumped from the mitochondrial matrix to the intermembrane space, creating an electrochemical gradient known as the proton-motive force (PMF), which comprises of an electrical component (Δψ) and a chemical component (ΔpH). Mitochondrial F1F0-ATP synthase (ATPase), also known as Complex V, uses PMF to generate ATP in the mitochondrial matrix (Mitchell, 1944; Spinelli and Haigis, 2018).

Mitochondria are highly dynamic organelles that maintain their shape, distribution, and size through coordinated cycles of fission and fusion known as mitochondrial dynamics (Giacomello et al., 2020). These processes, together with mitophagy (selective mitochondrial autophagy), maintain mitochondrial functionality during cellular stress conditions (Twig and Shirihai, 2011). Fusion helps mitigate stress by combining the contents of partially damaged mitochondria with those of fully functional ones, resulting in the formation of longer mitochondria. In contrast, fission facilitates quality control by promoting the removal of damaged mitochondria by mitophagy and triggering apoptosis during increased cellular stress (Chan, 2019). These continuous processes are crucial for maintaining mitochondrial homeostasis (Kiriyama and Nochi, 2018).

Arthropod vectors transmit some of the world’s most debilitating diseases, affecting millions of people, particularly in tropical and subtropical regions, which are home to half of the global human population. Arboviruses, or arthropod borne viruses, are maintained in nature through biological transmission between susceptible vertebrate hosts and hematophagous arthropods such as mosquitoes and ticks. Diseases such as Zika, Chikungunya, Yellow fever, and especially Dengue, are among the most epidemiologically and clinically significant arboviral diseases (World Health Organization, 2024). Moreover, mosquitoes’ dissemination is increasing, and vector control programs have thus far failed to halt the ongoing spread of arboviral diseases (Achee et al., 2019).

It is well established that viruses modulate the host cell by exploiting their molecular machinery to facilitate viral replication and propagation, including targeting mitochondrial functions and dynamics (Pando-Robles and Batista, 2017; Santana-Román et al., 2021; Whitley et al., 2019). During viral infections, mitochondria are directly targeted by viral proteins or indirectly affected by physiological alterations, such as oxidative stress, hypoxia, endoplasmic reticulum stress and dysregulated calcium homeostasis (Khan et al., 2015; Sousa et al., 2024). However, much of the current understanding of mitochondrial involvement during infection comes from studies conducted in mammalian cells. Relatively little is known about the alteration of mitochondrial function in arthropods during viral infection. Given this limited information, this review examines the existing literature on mitochondrial involvement in arthropod viral infections, comparing it with findings reported in mammal studies and other relevant non-viral infections in arthropods. Key gaps in the current field are identified, and critical questions for future research are highlighted.

### Metabolism in arthropods during arbovirus infection

Arboviruses have evolved to exploit the blood-feeding behaviors of their vectors, enabling the completion of their replication cycle in both humans and arthropods. While arboviral infections in humans are acute, they persist in mosquitoes throughout the insect’s lifespan. These infections impose fitness costs on mosquitoes, including reduced egg production, delayed hatching, and slower larval development (Chaves et al., 2019; Feitosa-Suntheimer et al., 2022). However, the metabolic responses to infection in invertebrates remain largely unexplored.

The dispersal capacity of insect vectors is critical for locating blood hosts, facilitating mating, spreading adaptive alleles between populations, and colonizing new ecological niches (Schmidt, 2025). In flying arthropods such as mosquitoes, flight muscles require high metabolic activity and energy expenditure (Mesquita et al., 2021). In *Aedes aegypti* (*Ae. aegypti*), the main transmitter of the dengue virus (DENV) and other arboviruses such as Chikungunya virus (CHIKV), yellow fever virus and Zika virus (ZIKV) (Weaver and Barrett, 2004), flight muscle activity and dispersal capacity are supported by mitochondrial function and cytochrome c oxidase (COX) (Gaviraghi et al., 2019). Flight muscles oxidize NAD^+^-bound substrates such as pyruvate and proline, as well as FAD^+^-bound substrates like glycerol 3-phosphate (G3P), as major sources of ATP production for vector dispersal (Soares et al., 2015). G3P oxidation in mitochondrial flight muscle is further modulated by adenylates through allosteric regulation of COX activity, with ADP acting as an activator and ATP as an inhibitor (Gaviraghi et al., 2019). Additionally, blood-feeding downregulates COX activity to prevent electron flow towards the production of ROS and harmful digestion byproducts like heme, which can damage cells (Gonçalves et al., 2009).

The G3P shuttle, which regenerates NAD^+^ in the cytosol and produces FADH_2_ in mitochondria, is essential for ATP production via OXPHOS (Hou, 2013). Two isoforms of G3P dehydrogenase have been identified in the flight muscles of *Triatoma infestans*, influenced by nutrition and rearing temperature (Stroppa et al., 2013). However, the role of the G3P shuttle in arthropod metabolism, and its potential impact on arboviral infections remain largely unexplored. Alterations in mitochondrial complexes are associated with decreased physical activity and diminished flight capacity in *Drosophila melanogaster (D. melanogaster)* and *Ae. Aegypti* (Gaviraghi et al., 2023; Rera et al., 2012). In mosquitoes, increased metabolic reserves are associated with prolonged flight durations, enhancing the probability of encountering multiple hosts (Carvajal-Lago et al., 2021). In female *Anopheles* mosquitoes, glycogen supports short flights, while fat reserves sustaining longer flight distances (Giulivi et al., 2017). Nevertheless, detailed metabolic changes related to dispersal capacity in arthropods during arbovirus infection remain unknown.

Arbovirus replication is closely linked to mitochondrial function (Table 1). Viruses such as Flock House virus (FHV) which infects *D. melanogaster,* and rice gall dwarf virus (RGDV) which infect *Nephotettix cincticeps*, rely on mitochondrial membranes for replication complex assembly (Stapleford et al., 2009; Wei et al., 2011). In the midgut of *Ae. aegypti* infected with DENV, glycerophospholipids are altered, and mitochondrial membranes are enriched of phosphatidylglycerol, a precursor for cardiolipin (CL) (Chotiwan et al., 2018). CL is a key component that stabilizes ETC complexes and prevents cytochrome c release (Paradies et al., 2019). Disruption of CL biosynthesis has been shown to impair DENV replication (Koh et al., 2020). CHIKV infection upregulates OXPHOS in the female *Ae. aegypti* during early infection (Cui et al., 2020; Shrinet et al., 2018). In *Ae. aegypti* Aag-2 cells infected with CHIKV, the mitochondrial ATPase gamma subunit and the Rieske subunit of ubiquinol-cytochrome b-c1 reductase (complex III) are upregulated, coinciding with the presence of abnormally elongated mitochondria (Vasconcellos et al., 2022). Similarly, Mayaro virus (MAYV) infection triggers the overexpression of the alpha subunit of ATP synthase in *Ae. Aegypti* (Vasconcellos et al., 2020).

**TABLE 1:** Metabolic changes during arbovirus infection in arthropods.

Arthropod	Arbovirus	Dysfunction	Consequence	References
*Aedes aegypti*	Dengue virus	Disruption of cardiolipin biosynthesis	Impairs DENV replication	Koh et al. (2020)
*Laodelphax striatellus*	Rice stripe virus	Upregulation of ATPase, MIT13 and NAD-dependent malic enzyme	Inhibition of TCA and OXPHOS activate compensatory pathways	Zhang et al. (2022)
*Aag-2 cells*	Chikungunya virus	Upregulation of complex III	Elongated mitochondria	Vasconcellos et al. (2022)
*Aedes aegypti*	Dengue virus	Upregulation of phosphatidylglycerol	Phosphatidylglycerol is a precursor of cardiolipin that maintain mitochondrial homeostasis	Chotiwan et al. (2018)
*Nephotettix cincticeps*	Rice gall dwarf virus	Mitochondrial degeneration	Assembles replication complex in the outer mitochondrial membrane mediated by P8 viral protein	Wei et al. (2011)
*Aedes aegypti*	Mayaro virus	Metabolic dysfunction	Upregulation of alpha subunit of ATP synthase	Vasconcellos et al. (2020)

Although multiple studies report changes in the expression of mitochondrial complex proteins during arthropod infections, their significance remains unclear (Cabezas-Cruz et al., 2017; Chisenhall et al., 2014; Pando-Robles and Batista, 2017). In mammalian cells, DENV infection induces alterations in ETC and OXPHOS parameters, increasing oxygen consumption linked to ATP production. The viral proteins NS4b and NS3 colocalize in the mitochondrial fraction associated with the endoplasmic reticulum (ER), though the functional implications of these interactions remain unclear (Barbier et al., 2017). Additionally, DENV infection impairs complex I, through the protease activity of NS3 viral protein, while leaving other respiratory complexes and maintaining MMP (Sousa et al., 2024).

Differences in mitochondrial function have been observed between *Anopheles gambiae* (*A. gambiae*) resistant and susceptible strains to *Plasmodium* infection. A resistant strain shows lower mitochondrial oxygen consumption, impaired state-3 respiration (maximal OCR with saturating ADP and substrates) increased electron leak and elevated ROS levels, without changes in maximum respiratory capacity. The diminished state-3 respiration is compensated by elevated glycolysis transcripts in the resistant strain, although these adaptations reduced the median lifespan of *A. gambiae* (Oliveira et al., 2011). Rice stripe virus (RSV) infection, transmitted by *Laodelphax striatellus* (*L. striatellus*), upregulates ATPase, mitochondrial import inner membrane translocase (MIT13), and NAD-dependent malic enzyme in its vector. Despite inhibition of these enzymes, RSV viral loads increase without compromising ATP production, suggesting the activation of a compensatory pathway, such as glycolysis to support RSV infection (Zhang et al., 2022).

Mitochondria also function as sensors in signaling pathways that regulate cell survival; however, the intrinsic mitochondrial apoptosis pathway in arthropods remains poorly studied. Lethal infection with *Anagrapha falcifera* multiple nuclear polyhedrosis virus triggers cytochrome c release from mitochondria, activating caspase 3, and reducing cell viability in *Spodoptera* cells (Liu et al., 2012). Similarly, white spot syndrome virus (WSSV) causes MMP loss in *Litopenaeus vannamei (L. vannamei)* hemocytes during late infection stages, promoting virion release and dissemination (Su et al., 2014). In the midgut of *Culex pipiens pipiens*, apoptosis serves as a regulatory mechanism to limit the dissemination of West Nile virus (WNV) to the salivary glands (Vaidyanathan and Scott, 2006). In *Ae. aegypti* midguts infected with DENV or ZIKV, early apoptosis is necessary to limit viral infection (Ayers et al., 2021). In DENV-infected C6/36 cells, elevated MMP, and sustained cell viability suggest a role for mitochondrial adaptations in supporting non-pathogenic viral persistence (Santana-Román et al., 2021).

The seemingly paradoxical roles of apoptosis in insect virus infection indicate a trade-off between infection control and pathological damage. These effects vary based on the specific virus-vector pairing (Ayers et al., 2021; Chen et al., 2011). In some cases, apoptosis following arbovirus infection correlates with refractoriness (Liu et al., 2013), while in others, it is associated with susceptibility and viral dissemination (Chen et al., 2011), ultimately influencing vector competence (O´Neill et al., 2015). Further research on the mitochondrial intrinsic apoptosis pathway is needed to clarify its role in shaping the arbovirus-arthropod relationship.

In mammalian cells, the loss of MMP is an early indicator of apoptosis during DENV and ZIKV infections (El-Bacha et al., 2007; Yang et al., 2020). DENV infection is associated with decreased MMP, increased oxygen consumption due to proton leakage, mitochondrial uncoupling, reduced ATP synthesis, and apoptosis induction (El-Bacha et al., 2007). Additionally, DENV infection impairs mitochondrial substrate oxidation, leading to reduced basal and maximal respiration (Fernandes-Siqueira et al., 2018).

Maintaining mitochondrial function and facilitating metabolic adaptations in infected cells are essential for viral persistence in invertebrates. Functional mitochondria play a pivotal role in the interactions between arboviruses and their arthropod vectors, influencing both viral propagation and host survival.

### The role of VDAC in arthropods during arbovirus infection

VDAC is a mitochondrial porin located in the OMM, playing a crucial role in communication between mitochondria and cellular organelles by regulating the transport of ions, ATP, and other metabolites. In mammals, three VDAC isoforms (VDAC1, VDAC2, and VDAC3) have been identified. The opening and closing of VDAC can influence mitochondrial OXPHOS and cytosolic ATP/ADP ratio, affecting the cell’s metabolic preference toward aerobic glycolysis (Maldonado and Lemasters, 2015; Najbauer et al., 2021). During apoptosis, VDAC interacts with pro-apoptotic proteins Bak and Bax, promoting OMM permeability facilitating cytochrome c release (Najbauer et al., 2021; Shoshan-Barmatz and Ben-Hail, 2012). In *Drosophila*, VDAC controls mitochondrial dynamics. Additionally, loss-of-function in VDAC is linked with flight disability and male infertility in *Drosophila* (Park et al., 2010).

**FIGURE 1:**
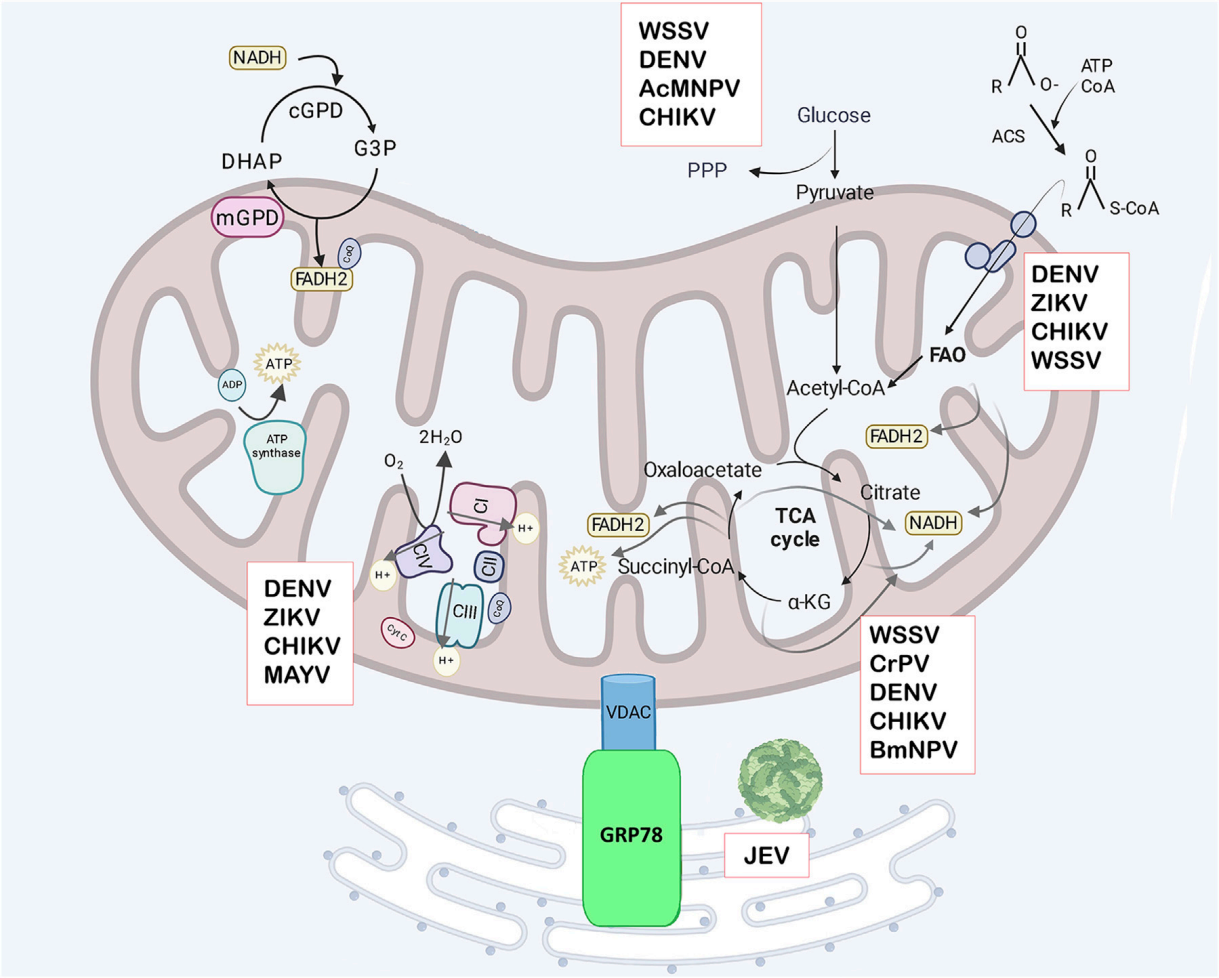
Mitochondrial metabolism. Glycerol-3 phosphate (G3P) production utilizes NADH in the cytosol and its oxidation generates FADH2 in the mitochondria, with the G3P shuttle acting as mechanism to regenerate cytosolic NAD+. The shuttle involves cytoplasmic G3P dehydrogenase (cGPD), and mitochondrial G3P dehydrogenase (mGPD) located on the outer face of the IMM. This FADH2 contributes to ATP generation via OXPHOS. The mitochondrial complexes are in the IMM, and transfer electrons in the electron transport chain (ETC) consisting of complexes (CI -CIV). Electron transfer produces an electrochemical gradient across the mitochondrial membranes which are used to produce ATP by ATP synthase in OXPHOS. Glucose from the cytosol can be diverted to the pentose -phosphate pathway (PPP) or converted to pyruvate in glycolysis and transported to the mitochondrial matrix where it is converted to Acetyl-CoA. Acetyl-CoA enters the tricarboxylic acid cycle (TCA), producing electron equivalents and ATP. Electron equivalents in the form of NADH or FADH2 are used in OXPHOS to produce ATP. Fatty acids are converted to acyl-CoA in the cytosol by acyl-CoA synthetase (ACS), then transported to mitochondria by the carnitine transporter and enter fatty acid oxidation (FAO) in the mitochondrial matrix, which produces Acetyl-CoA and electron equivalents. VDAC has been shown to have an important role in the viral replication cycle in arthropods. In mosquito cells infected with Japanese encephalitis virus (JEV), VDAC interacts with GRP78, suggesting that mitochondria relocate near the ER to supply ATP. Additionally, VDAC can promote or inhibit viral replication by regulating apoptosis in cells infected with different viruses. The boxes indicate the arboviruses that disrupt specific metabolic pathway. Created in Biorender.com. JEV, Japanese encephalitis virus, WSSV, white spot syndrome virus; DENV, dengue virus; ZIKV, Zika virus; CHIKV, Chikungunya virus; BmNPV, *B*. *mori* nuclear polyhedrosis virus; CrPV, Cricket paralysis virus, MAYV, Mayaro virus, and AcMNPV, *Autographa californica* multiple nuclear polyhedrosis virus.

In C6/36 mosquito cells infected with Japanese encephalitis virus (JEV), VDAC interacts with the ER protein GRP78 more frequently than in uninfected cells, suggesting mitochondria may relocate near the ER to supply ATP for viral replication (Figure 1) (Fongsaran et al., 2014; Romero-Brey I & Bartenschlager R, 2014). Additionally, VDAC re-localizes to the ER during DENV2 and DENV4 infection in mammalian cells, and its downregulation significantly reduced viral proteins levels, including E protein, NS1, NS3 and NS5 (Jitobaom et al., 2016). DENV NS4B also interacts with VDAC2 in a hepatic cell line, although the function of this interaction remains unknown (Chatel-Chaix et al., 2016). In arthropods, VDAC-pathogen interactions have also been observed. In the midgut of *Ripicephalus microplus*, the vector of *Babesia bigemina*, VDAC interacts with the parasite, and the infection induced VDAC upregulation (Rodríguez-Hernández et al., 2012).

VP7 from *Bombyx mori* cytoplasmic polyhedrosis virus (BmCPV) interacts with VDAC in the midgut of infected silkworms, indicating a potential role for mitochondria in BmCPV replication (L. He et al., 2017). In crustaceans, VDAC regulates WSSV infection by modulating the apoptosis pathway (Figure 1) (Gong et al., 2022; Wang et al., 2007). In *Procambarus clarkii*, VDAC overexpression reduces apoptosis in WSSV-infected hemocytes, whereas its downregulation decreases MMP, activates caspase 3, and suppress viral replication (Gong et al., 2022). Similar findings have been reported in *M. japonicus,* where VDAC inhibition reduces WSSV replication (Chen et al., 2011; Han-Ching Wang et al., 2010).

Conversely, in *L. striatellus*, VDAC2 upregulation (Figure 1) promotes RSV accumulation in midgut and salivary glands by activating apoptosis through caspases. This upregulation is also linked to interactions with viral RNA polymerase during infection, though its significance remains unclear (Zhang et al., 2023). In *Recilia dorsalis*, the non-structural protein Pns11 from RGDV interacts with VDAC to induce apoptosis via mitochondrial pathway, facilitating viral spread to salivary glands. Caspase inhibition reduces apoptosis and viral accumulation (Chen et al., 2019). Interestingly, in *Bombyx mori* (*B. mori*) strains resistant to *B. mori* nuclear polyhedrosis virus (BmNPV), VDAC is upregulated (Figure 1). In the BmN cell line, VDAC overexpression increases ROS production and caspase-3 activation, leading to reduced BmNPV replication by inducing apoptosis in infected cells (Lv et al., 2024).

These findings highlight the importance of VDAC regulation in balancing cell survival and viral replication. VDAC dysregulation can induce mitochondrial apoptosis, promoting or inhibiting viral infection (Figure 1). Targeting VDAC to prevent the transmission of *Babesia bigemina* has been proposed, and VDAC-related anti-tick vaccine are being studied for controlling tick infestations in cattle (Ortega-sánchez et al., 2019). Arthropods, including insects, arachnids, crustaceans and myriapods, often possess a single VDAC gene. However, in *Drosophila,* three VDAC isoforms exist due to duplication events (Graham and Craigen, 2005), and *L*. *striatellus* possesses two VDAC isoforms (Zhang et al., 2023). The amino acid sequence identity of VDAC between crustacean and insect is 59%–66%, while homology among crustaceans is 86%–92% (Gong et al., 2022; Han-Ching Wang et al., 2010). VDAC has also been identified in *A. gambiae*, the malaria vector, with 73% identity to *D. melanogaster* and 55% to human VDAC (Sardiello et al., 2003).

VDAC plays a multifaceted role in cellular metabolism and apoptosis, influencing viral pathogenesis in arthropods. Its evolutionary conservation and variations across arthropods emphasize its critical role in metabolism and pathogen interactions. Investigating VDAC in the *Culicidae* family, particularly in *Aedes* mosquitoes, may offer valuable insights into its functions and potential as a target for controlling arbovirus infections. Understanding these dynamics may reveal novel strategies for interrupting disease transmission and improving vector control.

### Energetic substrates and mitochondrial β-oxidation

Mitochondria plays a crucial role in fatty acid oxidation for energy production. Initially, fatty acids are converted into acyl-CoA in the cytosol by acyl-CoA synthetase (ACS). Acyl-CoA is then converted into acylcarnitine by the enzyme carnitine palmitoyltransferase 1 (CPT1) and transported into the mitochondrial matrix, where carnitine palmitoyltransferase 2 (CPT2) reconverts it into acyl-CoA. Within the mitochondria, acyl-CoA undergoes β-oxidation, a sequence of four enzyme-catalyzed reactions that produce acetyl-CoA, FADH_2_, and NADH. Acetyl-CoA enters the TCA cycle, while FADH_2_ and NADH donate electrons to ETC for ATP production (Figure 1) (Adeva-Andany et al., 2019).

Flight muscle of *Rhodnius prolixus* (*R. prolixus*) exhibit higher fatty acid oxidation rates compared to other tissues (De Paula et al., 2023). In female *Ae. aegypti*, β-oxidation supports the gonadotrophic cycle (Wang et al., 2017). Acetyl-CoA, a product of fatty acid oxidation, is redirected toward the synthesis of amino acids and polyamines, ensuring egg viability during desiccation stress. CPT1 inhibition significantly reduces egg viability under stress conditions in *Ae. aegypti* (Prasad et al., 2023). Similarly in *R. prolixus,* knockdown of ACSL2, a long-chain acyl-CoA synthetase, reduces β-oxidation, leading to decreased oviposition and egg hatching (Alves-Bezerra et al., 2016). Interestingly, while CPT1 inhibition does not directly impair β-oxidation, it disrupts lipid mobilization and starvation resistance in *R. prolixus* (De Paula et al., 2023). In contrast, inhibition of the Mitochondrial Trifunctional Protein A Subunit minimally affects oviposition but reduces flight capacity in *R. prolixus* (Aredes et al., 2022). These findings highlight the importance of β-oxidation and its compensatory mechanisms in supporting arthropod vector development, reproduction and adaptability to environmental and nutritional stresses (Alves-Bezerra et al., 2016; Aredes et al., 2022; De Paula et al., 2023; Prasad et al., 2023)

Lipid biosynthesis and redistribution of them are important for arbovirus replication (Hsieh et al., 2015; Perera et al., 2012; Vial et al., 2019). Nevertheless, the implication of β-oxidation pathway during arbovirus replication in arthropods needs to be explored, since there are few examples in the literature. Acyl-carnitines, which transport fatty acyl-CoA from the cytoplasm to the mitochondria, were found to increase in the midgut of *Ae*. *aegypti* and in Aag2 cells following DENV and ZIKV infection (Chotiwan et al., 2018; Manokaran et al., 2020). In *Wolbachia*-infected *Ae. Aegypti*, downregulation of acyl-carnitines reduces DENV and ZIKV replication, but supplementation of acyl-carnitines restores flavivirus replication (Manokaran et al., 2020). In DENV-infected *Ae. aegypti*, β-oxidation enzymes such as 3-Ketoacyl-CoA thiolase and 3-hydroxyacyl-CoA dehydrogenase are downregulated (Shrinet et al., 2018). Similarly, acyl-CoA dehydrogenase is downregulated in CHIKV-infected mosquitoes (Cui et al., 2020). In *L. vannamei*, increased expression of fatty acid synthase and lipogenesis during late-stage infection supports viral particle assembly. However, the inhibition of CPT1 decreases WSSV production (Hsieh et al., 2015). Additionally, WSSV-infected shrimp experience a reduction in TAG content during viral genome replication, suggesting that maintaining β-oxidation pathway is crucial during infection (Chen et al., 2011).

In mammals, DENV infection induces the processing of lipid droplets by autophagy, to release free fatty acids for β-oxidation. Inhibiting autophagy reduces DENV replication due to decreased β-oxidation (Heaton and Randall, 2011; Rasmussen et al., 2011). While fatty acids serve as main energy source during DENV infection (Fernandes-Siqueira et al., 2018), JEV inhibits β-oxidation via the NS5 viral protein (Kao et al., 2015). Both insect and mammalian cells remodel intracellular membrane during viral replication, requiring fatty acids rerouted from β-oxidation to lipid synthesis (Junjhon et al., 2014). Inhibition of fatty acid synthase diminished the WNV and Usutu virus replication (Martín-Acebes et al., 2011). ZIKV alters host lipid composition through the NS4B viral protein, and disruption of sphingolipid biosynthesis inhibits ZIKV infection in human neural progenitor cells (Leier et al., 2020).

While β-oxidation is important in some viral contexts, arboviruses may suppress this pathway to promote lipid synthesis for replication and assembly. Further research is needed to clarify β oxidation´s role during arbovirus infection in arthropods.

### TCA cycle and anaplerotic routes during infection

The TCA cycle consists of a series of chemical reactions that release stored energy by oxidizing acetyl-CoA derived from carbohydrates, fatty acids, or proteins (Figure 1). Most of the energy from the TCA cycle is captured by the oxidized NAD^+^ or FAD, which transfer their electrons to the ETC, leading to ATP production through OXPHOS (Martínez-reyes and Chandel, 2020). When TCA cycle intermediates are depleted, anaplerosis replenishes them, maintaining the cycle´s function and supporting cellular metabolism. Pyruvate carboxylase (PC) is a key enzyme in anaplerosis, catalyzing the carboxylation of pyruvate to produce oxaloacetate (Giulivi et al., 2017; Owen et al., 2002). During aerobic glycolysis, or the Warburg effect, glucose carbons are diverted away from the TCA cycle, with glutamine playing a crucial role in replenishing the cycle to support mitochondrial oxidative metabolism, known as glutaminolysis. Glutaminase (GLS) hydrolyzes glutamine into glutamate, that can be converted into α-ketoglutarate by glutamate dehydrogenase (GDH) (Russell and Taegtmeyer, 2013; Yoo et al., 2020). TCA cycle is fundamental to arthropod survival, influencing both their metabolic state and response to environmental challenges. In *Helicoverpa armigera,* diapause is induced by reduction of the TCA cycle activity, while increased activity promotes growth and development (Wang et al., 2018). In some hymenopterans, proline serves as a carbon source to replenish TCA cycle intermediates, sustaining flight activity (Teulier et al., 2016).

In *Ae. aegypti* salivary glands infected with DENV, overexpression of isocitrate dehydrogenase and pyruvate dehydrogenase, key enzymes linking glycolysis to the TCA cycle have been detected (Chisenhall et al., 2014). Similarly, in Aag2 cells infected with CHIKV, CoA biosynthesis is upregulated, supporting both TCA cycle and β-oxidation (Mehta et al., 2024). In *B. mori* infected with BmNPV, citrate synthase (CS) is upregulated, enhancing ATP production and MMP, whereas inhibiting CS activity reduces viral replication (Hu et al., 2022). However, in infected *B. mori* larvae, glutaminolysis plays a minor role in BmNPV replication, with amino acid and lipid metabolism upregulation (Feng et al., 2021).

During the transition from persistent to pathogenic infection in Cricket paralysis virus (CrPV) in *B. mori* Bm5 cells, significant changes in amino acid metabolites occur, particularly a decline in glutamine during the pathogenic stage (Wang et al., 2019). As a non-enveloped virus, CrPV, relies on glutamine for viral protein synthesis rather than TCA cycle replenishment or lipid biosynthesis (Fendt et al., 2013). In contrast, WSSV, an enveloped virus, increases citrate levels to support lipid biosynthesis during viral production (He et al., 2019). In *L*. *vannamei* infected with WSSV, aerobic glycolysis is activated, diverting glucose to pyruvate while acetyl-CoA is redirected towards lipid biosynthesis via citrate, instead of entering the TCA cycle (Su et al., 2014). WSSV also modulates key glutaminolysis enzymes to replenish the TCA cycle (Li et al., 2016; Tan et al., 2024). In infected shrimp, increased glutamate uptake via the SLC1A2 transporter and enhanced GDH and aspartate aminotransferase (ASAT) activity drive glutamate-mediated anaplerosis, although GLS expression remain unchanged (He et al., 2019; Li et al., 2016). The upregulation of α-ketoglutarate dehydrogenase (α-KGDH) and isocitrate dehydrogenase 1 (IDH1) supports WSSV replication, while inhibiting these enzymes reduces it (He et al., 2019). WSSV infection also enhance GDH activity via SIRT4, facilitating the conversion of glutamate to α-ketoglutarate, further boosting viral genome replication (Tan et al., 2024).

A metabolomic analysis of patients with DENV and CHIKV mono-infections or co-infections reveals alterations in TCA intermediates compared to healthy individuals (Shahfiza et al., 2017; Shrinet et al., 2016). Similarly, symptomatic yellow fever virus infections are associated with reduced TCA cycle activity compared to asymptomatic cases, characterized by lower levels of citrate, isocitrate and malate, suggesting that TCA cycle activity may influence susceptibility to symptomatic infection (Chan et al., 2019). In mammalian cells infected with DENV, glutamine depletion minimally affects oxygen consumption and viral replication compared to glucose deprivation, suggesting that glutamine plays primarily a biosynthetic role rather than an oxidative one (Fernandes-Siqueira et al., 2018; Fontaine et al., 2015). In hepatic cell lines, DENV and ZIKV infections alter subunits of complex II (succinate dehydrogenase), a component of both TCA cycle and the ETC. These alterations correlate with significant reductions in TCA metabolites, including α-ketoglutarate and succinate for ZIKV and fumarate and malate for DENV. Conversely, DENV infection increases citrate and cis-aconitate levels, further implicating TCA cycle dysfunction in flavivirus infections (Freppel et al., 2025).

While the TCA cycle is not universally essential for viral replication, its modulation and the resulting metabolite changes play critical roles in infection dynamics, underscoring the complexity of host-pathogen interactions.

### Glycolysis pathway and Warburg effect on infection

Glycolysis is a highly conserved metabolic pathway that occurs in the cytoplasm of cells, where one glucose molecule is converted into two pyruvate molecules (Figure 1), generating two ATP molecules. Pyruvate is transported into the mitochondrial via pyruvate carriers (MPC) and enters the TCA cycle. The complete oxidation of one glucose molecule via mitochondrial OXPHOS produces 36 ATP molecules. In arthropods, glycolysis generates intermediates that fuel biosynthetic pathways essential for growth, and development during the larval stage, and metamorphosis (Chandel, 2021; Da-Ré et al., 2014; Zhao et al., 2022).

In *Ae. Aegypti* salivary glands infected with DENV and in infected C6/36 cell line, glycolytic enzymes such as phosphoglycerate kinase, enolase, and fructose bisphosphate aldolase are upregulated (Chisenhall et al., 2014; Patramool et al., 2011). In *Ixodes scapularis* (*I. scapularis*) midgut infected with *Anaplasma phagocytophilum* (*A. phagocytophilum*), glucose transporters are upregulated, while glucose 6-phosphatase is downregulated retaining glucose 6-phosphate to be metabolized in the cell for pathogen proliferation (Cabezas-Cruz et al., 2017). *In vitro* models of *A. phagocytophilum* infection show a preference for glycolysis over gluconeogenesis. Reduced PEPCK protein levels in infected *I*. *scapularis* ISE6 cells, lower gluconeogenesis, reducing apoptosis and enhancing infection, while PEPCK activation increased apoptosis and limits infection (Villar et al., 2015). These finding suggests that glycolysis modulates pathogen transmission, with the salivary glands and midgut playing key roles in pathogen dissemination (Franz et al., 2015).

Beyond energy production, glucose metabolism influences pathogen replication and cellular viability. The interplay between host carbohydrate metabolism and infection appears integral to insect permissiveness and persistence, with insulin signaling contributing to metabolic regulation (Saltiel, 2021). For instance, females of *D. melanogaster* Dicer-2 mutants exhibit altered insulin signaling and decreased carbohydrate metabolism, while *D. melanogaster* mutants for the chico gene (insulin receptor substrate 1) are more susceptible to ZIKV infection due to the downregulation of the RNA interference pathway, highlighting the role of insulin signaling in countering infection (Tafesh-Edwards et al., 2022). In *B. mori*, glycolysis and ATP production increase during non-permissive infection with *Autographa californica* multiple nuclear polyhedrosis virus (AcMNPV). Suppressing glycolysis enhanced AcMNPV replication by reducing the expression of the antibacterial peptide gloverin (Lin et al., 2020). In *B. mori* BM5 cells infected with CrPV, glucose and glutamine levels increased during persistent infection and decreased during acute pathogenic stages, suggesting glycolysis modulates infection (Wang et al., 2019).

During infection, metabolic changes often resemble the “Warburg effect,” where cells prioritize glycolysis over OXPHOS, even with sufficient oxygen and functional mitochondria. Initially identified in cancer cells (Chandel, 2021), this phenomenon, also known as aerobic glycolysis, occurs in both vertebrate and invertebrate cells during infection (Krejčová et al., 2019; Pouyssegur et al., 2022). The Warburg effect supports rapid production of biosynthetic precursors and electron carriers like NADPH, essential for anabolic and redox reactions (Liberti et al., 2017; Yoo et al., 2020). Pyruvate from glycolysis helps to maintaining the NAD^+^/NADH balance via lactate dehydrogenase (Lin et al., 2022), while glycolytic intermediates fuel the pentose phosphate pathway (PPP) producing ribose-5-phosphate for nucleotide synthesis and NADPH for reduction reactions (Stincone et al., 2015).

In CHIKV- or DENV-infected *Ae. Aegypti,* upregulation of fructose-1,6-biphosphatase redirects glucose to the PPP for ribose production, essential for viral genome replication (Shrinet et al., 2018). Similarly, WSSV infection in shrimp hemocytes induces the Warburg effect, increases glucose-6-phosphate dehydrogenase (G6PDH) activity and reroutes glucose to the PPP (Chen et al., 2011; Su et al., 2014). G6PDH is a key enzyme in the first step of PPP, converting glucose-6 phosphate (G6P) to 6-phosphogluconolactone (Chandel, 2021). ZIKV infection in C6/36 mosquito cells enhances PPP pathway, unlike in human cells, where infection favors the TCA cycle and reduces PPP flux, leading to ATP depletion, AMPK activation and caspase 3- mediated cell death. Inhibiting G6PDH in C6/36 cells mimics the response seen in human cells, highlighting metabolic differences between species (Thaker et al., 2019).

The Warburg effect is regulated by hypoxia-inducible factor 1 (HIF-1α), which is activated by growth factors, hypoxia, and infections (Lu et al., 2002; McGettrick and O’Neill, 2020; van Uden et al., 2011). The gut microbiota induces era hypoxic environment, activating HIF-1α to promote growth in Ae*. aegypti* larvae, while normoxic conditions and the HIF-1α inhibitors suppress larval growth (Valzania et al., 2018). Infected *I. scapularis* with *A. phagocytophilum* exhibits a Warburg-like phenotype driven by HIF-1α via PI3K pathway (Cabezas-Cruz et al., 2017). The PI3K-Akt-mTORC1 pathway similarly triggers the Warburg effect in WSSV-infected shrimp hemocytes (Su et al., 2014). In *Drosophila* hemocytes, HIF-1α activation during acute *Staphylococcus pneumoniae* infection increases glycolytic flux, which returns to baseline after infection resolves (Krejčová et al., 2019). Also, chemical inhibition of pyruvate dehydrogenase kinase 1 (activate pyruvate dehydrogenase) redirects metabolism from glycolysis to OXPHOS, enhancing survival during lethal infections (Bakalov et al., 2020). Persistent glycolysis and lactate production during infection can be harmful, and shifting back to OXPHOS is crucial for survival and disease tolerance (Ayres and Schneider, 2015).

In ZIKV-infected human endothelial cells, increased glycolysis and ATP production are associated with upregulated glucose transporter 1 (GLUT1) and glycolytic genes such as hexokinase 2 (HK2), triosephosphate isomerase (TPI), and monocarboxylate transporter 4 (MCT4). Besides, AMPK activation or glycolysis inhibition reduces viral replication (Singh et al., 2020), and similar inhibition decreases WNV replication (Mingo-Casas et al., 2023). During DENV infection in human hepatic cells under short-term starvation, glucose is diverted for anaplerosis, favoring β-oxidation of fatty acids as a primary energy source (Fernandes-Siqueira et al., 2018). This aligns with DENV infection kinetics, where glycolytic intermediates accumulate differently depending on the viral replication stage, as viral proteins interact with glycolytic enzymes to modulate this pathway (Allonso et al., 2015; Fontaine et al., 2015; Silva et al., 2019; Vasconcellos et al., 2020). In insects, viral proteins like CrV1 from *Cotesia plutellae* bracovirus regulate glycolysis to suppresses immune responses, illustrating the broader impact of glycolysis on infection (Kumar and Kim, 2016).

Understanding the regulation of glycolysis is crucial for explaining differences in host permissiveness and lethal infections in arthropods vectors. However, significant knowledge gaps remain regarding metabolic response in insects, warranting further investigations to clarify regulatory mechanisms.

### Mitochondrial ROS and protein carriers

Reactive oxygen species (ROS) can be produced in the cytoplasm by NADPH oxidases, but the mitochondria are the primary source (Nathan and Cunningham-Bussel, 2013). Mitochondrial complexes I and III are key producers of ROS (Willems et al., 2015). ROS, including superoxide anions (O_2_^.-^) and hydrogen peroxide (H_2_O_2_), function as signaling molecules in innate immune response, cell proliferation, differentiation, and stress responses (Nathan and Cunningham-Bussel, 2013). Under normal conditions, a small fraction of electrons leak from the ETC, interacting with oxygen to produce superoxide (Brookes, 2005). Complex I generate ROS during the transfer of electrons from NADH to coenzyme Q (CoQ), while complex III produces ROS due to CoQ cycle inhibition, leading to the accumulation of semiquinone (Q^−^), which reduces O_2_, generating O_2_^−^ (Zhao et al., 2019). Then, mitochondrial superoxide dismutase converts O_2_^.-^ to H_2_O_2_ (Murphy, 2009). ROS production is tightly controlled by proton leak, which occurs when a small number of protons bypass ATP synthase and flow directly into the mitochondrial matrix without generating ATP. Uncoupling proteins (UCPs) and adenine nucleotide translocator (ANT) regulate proton leak, influencing ROS production and mitochondrial respiration (Slocinska et al., 2016). Proton leak diminishes ROS production, while ROS can induce proton leak, creating a feedback regulatory loop (Brookes, 2005)

Intracellular ROS are generated through oxidative phosphorylation in the mitochondria and by NADPH oxidases, playing diverse roles in insect physiology, life cycle and survival (Bottino-Rojas et al., 2018; Geng et al., 2021; Legan et al., 2008). In arthropods vectors, maintaining a balance between ROS production and neutralization is essential for vector competence. Disruption of this balance can lead oxidative stress, resulting in cell death (Oliveira et al., 2017). For instances, exogenous H_2_O_2_, induces oxidative stress in *B. mori*, causing mitochondrial depolarization, cytochrome c release, and apoptosis (Chen et al., 2015). A heterozygous OPA1 mutation increases ROS, impairing mitochondrial complexes II and III, reducing aconitase activity, and shortening the lifespan of *D. melanogaster* (Tang et al., 2009). In the flight muscle of *Ae. aegypti*, blood digestion activates mitochondrial fusion (increased Mfn and OPA1 levels) and reduces complex IV activity, thereby diminishing ROS production as a mechanism to mitigate oxidative stress (Gonçalves et al., 2009). These examples underscore the complex role of mitochondrial ROS in arthropods, including their influence on mitochondrial dynamics and the regulation of metabolic enzymes.

ROS and antioxidant system influence arthropod defenses against infections, potentially mediating resistance or persistent/tolerant infections (Mehta et al., 2024; Molina-Cruz et al., 2008; Selot et al., 2007; 2010). In DENV-infected C6/36 cells the unfolded protein response (UPR) pathway alleviates MMP loss and reduces ROS production, maintaining cell viability (Chen et al., 2017). In ticks, mitochondrial ROS limit bacterial infection. In *I. scapularis,* elevated ROS production is associated with increased complex I and complex III activity and reduced antioxidant system in midgut and salivary glands, leading apoptosis and limiting *A*. *phagocytophilum* infection (Alberdi et al., 2019). Similarly, *A. gambiae* mitochondrial changes during infection promote ROS production. Inhibition of ANT increases midgut H_2_O_2_ production, reducing *Plasmodium* prevalence, while silencing mitochondrial carrier 1 (MC1) increases proton leak and decreases midgut ROS production, increasing susceptibility to infection (Gonçalves et al., 2012; Oliveira et al., 2011). In non-infectious conditions, UCPs regulate mitochondria uncoupling and ROS production. For example, in *R*. *prolixus*, UCP4 expression in midgut enterocytes after a blood meal reduces ROS production, preventing oxidative stress. Inhibition of UCP4 activity increases H_2_O_2_ generation (Alves-Bezerra et al., 2014). In *D. melanogaster*, UCP4C mediates uncoupled respiration during larval stage, facilitating cold adaptation (Da-Ré et al., 2014). These findings suggest UCPs play a critical role in arthropod adaptations by modulating mitochondrial function, though their role in arbovirus infection remains unknown. In shrimp (*Fenneropenaeus indicus)*, the translationally controlled tumour protein enhances hemocyte survival by preserving MMP and reducing ROS levels, thereby inhibiting mitochondrial apoptosis to allow WSS clearance by hemocytes (Rajesh et al., 2014). In *L. vannamei* infected with *Vibrio parahaemolyticus*, upregulation of sirtuin 6 helps maintain MMP, decreasing ROS generation, inhibiting cytochrome c release, and preventing hemocyte apoptosis (Nie et al., 2020).

In mammalian cells infected with ZIKV, mitochondrial ROS generation activates apoptosis (Frumence et al., 2016). At later stage of infection, ZIKV induces mitochondrial impairment and ROS production, leading to DNA damage (Ledur et al., 2020). Similarly, DENV infection causes mitochondrial membrane leak, and a reduction in MMP, resulting in elevated mitochondrial ROS levels and loss of endothelial permeability (Meuren et al., 2022). Venezuelan Equine Encephalitis Virus reduces mitochondrial activity and increased ROS in an astrocytoma cell line, although, in C6/36 mosquito cells do not show significant decreases in MMP or substantial ROS increases, indicating they may be less susceptible to mitochondrial disruption compared to human cells (Keck et al., 2017).

ROS function as critical second messengers that mediate various intracellular pathways (R. U. Z. Zhao et al., 2019). For example, the activation of ATPase Inhibitory Factor 1 induces mitochondrial O_2_^.-^ production, which subsequently activates NF-κB, initiating the transcription of the WSSV genome (Huo et al., 2020). In *Ae. aegypti* and *Ae. albopictus* cells infected with DENV, p53 is upregulated in response to ROS, facilitating viral dissemination and promoting cell survival by regulating catalase expression. However, the role of mitochondrial ROS in the activation of p53 remains unknown (T. H. Chen et al., 2018).

Vector competence, which defines a vector’s ability to acquire, host, and transmit a pathogen, relies on maintaining a delicate balance of ROS levels. This balance supports the entry, survival, and proliferation of pathogens within the vector while ensuring the arthropod’s survival and feeding behavior, ultimately facilitating pathogen transmission (Hernandez et al., 2022). Thus, ROS plays a complex role in arthropod defense against infections. Elevated ROS levels, regulated by mitochondrial proteins and carriers, can enhance infection resistance, while improper regulation can increase susceptibility.

### Mitochondria dynamics

Changes in the morphology, quantity and position of mitochondria within eukaryotic cells are collectively referred to as mitochondrial dynamics, which are essential for proper cellular function (Giacomello et al., 2020). Current understanding of mitochondrial dynamics primarily originates from studies in yeast, mammals, and fruit flies, particularly in the relation to their impact on human health (Whitley et al., 2019). However, this field remains underexplored in medically important arthropods vectors of pathogens, such as mosquitoes. Mitochondrial dynamics play a critical role in cellular metabolic flexibility, as mitochondria continuously undergo cycles of fission, fusion, mitophagy, and transport. These processes allow for the removal of damaged components or impaired mitochondria through mitophagy, preventing cellular damage. Maintaining a balance in these dynamics is crucial for optimal mitochondrial function and overall cell health (Giacomello et al., 2020; Willems et al., 2015).

**FIGURE 2:**
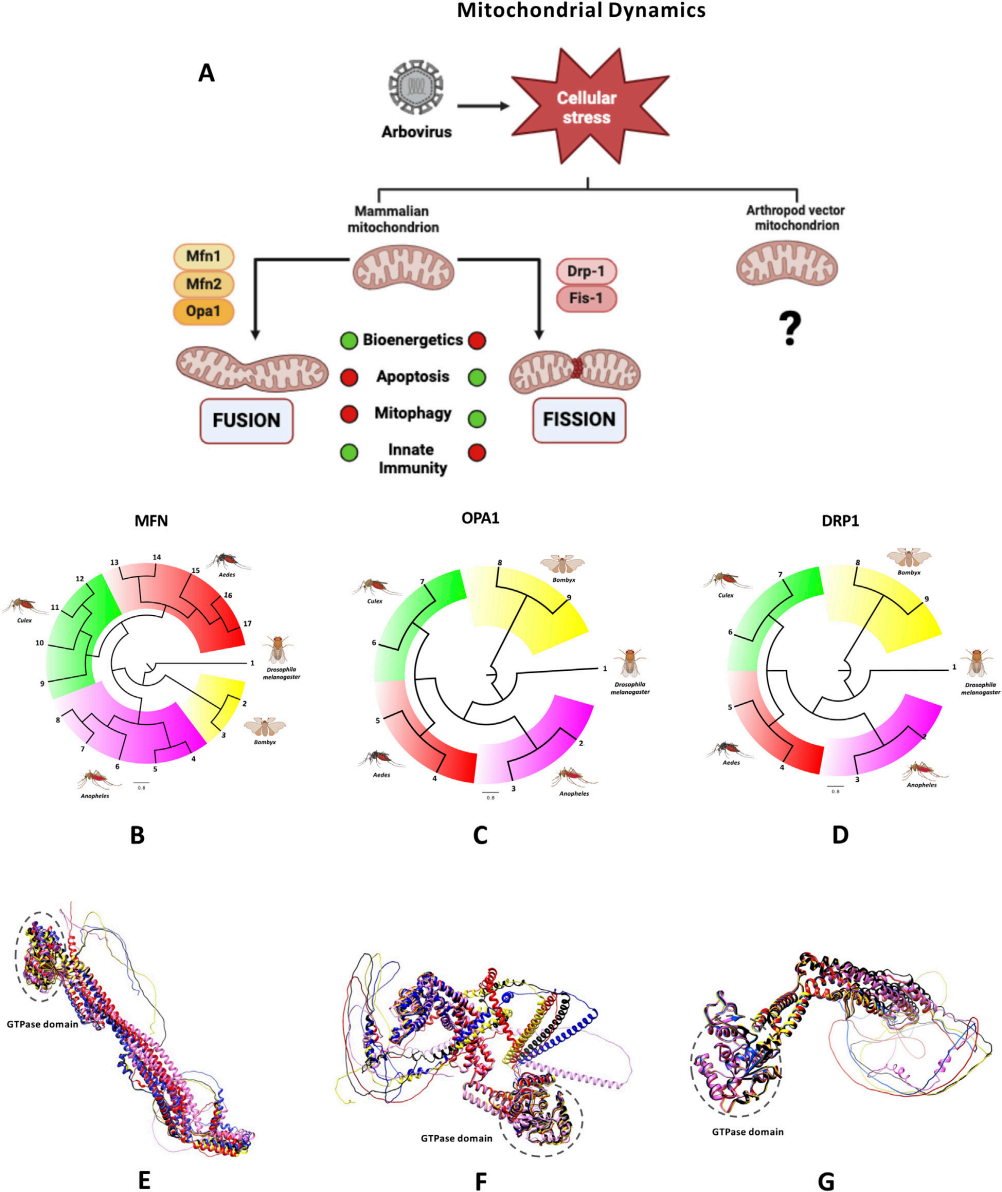
Mitochondrial dynamics-related proteins. **(A)** Mitochondria are dynamic organelles that constantly undergo fusion and fission. This figure illustrates the proteins involved in mitochondrial dynamics in mammals while emphasizing the limited data available for arthropods. Arbovirus infection induces cellular stress, disrupting the balance between fusion and fission, which impairs key cellular processes. These processes are represented using a traffic light, with green indicating normal functionality and red signifying inhibition. Created in BioRender.com. **(B–G)** Key proteins involved in mitochondrial fusion and fission are illustrated. Panels **(B–D)** present phylogenetic trees based on amino acid sequences of Mfn-like, Opa1-like, and DRP1-like proteins from relevant arthropods of the genera Aedes, Culex, and Anopheles, as well as Bombyx mori were generated using MAFFT software (v7.511). Corresponding proteins from Drosophila melanogaster serve as the outgroup. The trees are scaled, with branch lengths representing the evolutionary distances. Numbers represent the sequences used to generate the trees. Groups are color-coded using FigTree (v1.4.4). Panels **(E–G)** present structural alignments for Mfn-like sequences, Opa1 and DRP1, in all cases, the structures of Drosophila melanogaster are shown in red, Aedes aegypti in black, Culex pipiens in yellow, Anopheles albimanus in blue and Bombyx mori in pink. In all alignments, the GTPase domain is highlighted in dotted circles. Protein sequences were modeled using the ColabFold program (v1.5.5).

Mitochondrial dynamics, characterized by the balance between fusion and fission (Figure 2), are regulated by dynamin-related proteins located in both the IMM and OMM. These proteins share a highly conserved GTPase domain, enabling self-assembling, GTP hydrolysis, and membranes remodeling. Key proteins involved in mitochondrial fusion include mitofusins 1 and 2 (Mfn1 and Mfn2) and optic atrophy protein 1 (OPA1). Fusion allows mitochondria to merge their outer and inner membranes, facilitating the exchange of mitochondrial DNA, proteins, and metabolites. The fusion of OMM is controlled by Mfn1 and Mfn2, which form both homotypic and heterotypic dimers (Koshiba et al., 2004). IMM fusion is regulated by OPA1, which is processed by the metalloproteases OMA1 and YME1L, creating long (L-OPA1) and short (S-OPA1) forms. Any imbalance in these isoforms impairs the mitochondrial fusion mechanism (Head et al., 2009; Song et al., 2007).

Mitochondrial fission, in contrast, is mediated by dynamin-related protein 1 (DRP1), which is recruited from the cytosol to mitochondrial constriction sites. DRP1 oligomerizes around mitochondria, and GTP hydrolysis induces conformational changes that drive the fission of both the OMM and IMM. DRP1 recruitment is regulated by various post-translational modifications, such as phosphorylation, ubiquitination, and SUMOylation (Hu et al., 2017). Specific OMM receptors, including mitochondrial fission factor (MFF), mitochondrial fission protein 1 (FIS1), and mitochondrial dynamic proteins of 49 and 51 kDa (also known as MIEF2 and MIEF1), facilitate Drp1 targeting (Losón et al., 2013; Palmer et al., 2013). Genetic loss of DRP1 results in a dramatic mitochondrial elongation (Ishihara et al., 2009; Smirnova et al., 2001; Wakabayashi et al., 2009).

Mitophagy starts with the sensing of low cellular energy levels by AMPK. This signaling, combined with the loss of MMP, constitutes the typical red flag that triggers the molecular cascade leading to mitochondrial clearance. This process can be initiated by two pathways, one dependent and another independent on ubiquitination. A conserved step across these pathways involves interaction between mitochondrial cargo receptors (MCR) and microtubule-associated proteins 1A/1B-light chain 3 (LC3), which facilitates autophagosome binding. To initiate mitophagy, Drp1 mediates mitochondrial fission, separating damaged mitochondria from the healthy population. The ubiquitination-dependent pathway is mediated by PINK1/Parkin. PINK1 is a serine/threonine kinase and Parkin is an E3 ubiquitin ligase that adds ubiquitin molecules to OMM proteins. Ubiquitination-independent mitophagy involves stress-induced MCRs such as FUNDC1, BNIP3, NIX, and Bcl2-L-13. These receptors locate to the OMM and possess an LC3 interacting region (LIR) motif that bind to LC3 and recruit autophagosomes (Novak et al., 2010; Schwarten et al., 2009).

In arthropod vectors like mosquitoes, mitochondrial dynamics and associated proteins remain poorly understood. Mitochondrial fusion has been observed in *Ae*. *aegypti* flight muscle following blood digestion, with increased expression of the Mfn and OPA1 genes (Gonçalves et al., 2009). Our group has also shown Mfn and UCP2 colocalization, suggesting mitochondrial fusion in C6/36 cells infected with DENV2 (Santana-Román et al., 2021). In Aag-2 cells infected with CHIKV, mitochondria exhibit an elongated morphology, though the role of fusion during infection remains unclear (Vasconcellos et al., 2022).

By searching publicly available mosquito sequence databases, we identified homologous sequences for Mfn, Opa1, and Drp1 proteins in various mosquito species, including *Ae. aegypti*, *Ae. albopictus*, *A. gambiae*, and *Culex pipiens*. These mitofusin-like proteins show a high sequence identity (80%–99%) but lower identity (33%–40%) when compared to *D. melanogaster* (Uniprot: O18412). For DRP1 homologs, a single sequence was identified in each of the mosquito species. DRP1 homologs display 78%–81.5% identity to the *Drosophila* sequence, while OPA1-like proteins show 74%–77% identity. Phylogenetic trees generated using the neighbor-joining method confirm the close relationships between these mosquito proteins and their counterparts in other arthropods. Structural modeling indicates that despite the lower identity with *Drosophila*, mosquito mitofusins retain a conserved structural fold, essential for their function in eukaryotic cells. In summary, Mfn-like, Drp1-like, and Opa1-like proteins in mosquitoes share a high degree of amino acid sequence identity and exhibited close phylogenetic relationships, as illustrated by the phylogenetic trees generated from these sequences (Figures 2B–D). Although mosquito Mfn-like proteins display lower identity compared to Mfn sequences from other arthropods such as *D. melanogaster* and *B. mori*, they retain a conserved structural fold, consistent with their essential and evolutionarily conserved function in eukaryotic cells (Figure 2E–G).

Understanding the structure and function of these proteins in mosquitoes is critical for developing molecular tools to study mitochondrial dynamics during infection. Commercial antibodies that recognize proteins in animal models (mouse, *Drosophila*) or human, often fail to recognize mosquito proteins, so custom antibodies are needed. Developing such tools is essential for studying mitochondrial dynamics and their role in arbovirus infection in mosquitoes.

Mitophagy in arthropod vectors during infection remains poorly characterized, though emerging evidence highlights its significance. The southern rice black-streaked dwarf virus, an insect-borne plant reovirus, induces mitophagy in its vector *Sogatella furcifera*. The viral protein P7-1 localizes to the mitochondria, activating BNIP3 dimerization, which interacts with ATG8/LC3, promoting mitochondrial sequestration into autophagosomes and inhibit apoptosis (Liang et al., 2023). Similarly, RGDV, transmitted by *Recilia dorsalis*, induces mitophagy via the viral protein Pns11, which depends on VDAC1 channel as a docking site for autophagosome formation. The balance between mitophagy and apoptosis is critical for maintaining mitochondrial quality control and ensuring viral persistence in insect vectors (Chen et al., 2023).

In mammals, pathogens can manipulate mitochondrial dynamics to enhance their proliferation (Ren et al., 2020). For example, DENV infection, inhibits DRP-1 activation (phosphorylation) via the nonstructural protein NS4B, promoting mitochondrial elongation and viral replication (Chatel-Chaix et al., 2016). Conversely, inducing mitochondrial fission through DRP-1 overexpression or a mitochondrial uncoupler (carbonyl cyanide m-chlorophenylhydrazone) reduces viral replication (Barbier et al., 2017). Similarly, ZIKV NS1 protein induces mitochondrial fragmentation and cell death in neurons (Yang et al., 2020) and increasing fission in retinal pigmented epithelial cells (Russo et al., 2021). Infection with Venezuelan equine encephalitis virus alters mitochondrial dynamics in astrocytes, inducing mitophagy mediated by the re-localization of PIKNI and Parkin to the mitochondrial membrane. Treatment with a mitochondrial fission inhibitor decreases caspase cleavage, suggesting that Venezuelan equine encephalitis virus contribute to apoptosis through a mechanism dependent on mitochondrial disruption (Keck et al., 2017).

Viruses can also induce mitophagy to prevent apoptosis and inhibit mitochondria-dependent immune signaling, facilitating viral propagation and persistence (Khan et al., 2015). For instance, BNIP3 modulates CHIKV infectivity; knockdown of this protein increases the number of infected cells, suggesting that BNIP3 interferes with CHIKV replication independently of its role in mitophagy and mitochondrial homeostasis (Echavarria-Consuegra et al., 2023). Additionally, ZIKV infection in trophoblast cells induces mitophagy through NS4A protein to evade immune signaling and promote viral propagation (Lee and Shin, 2023).

Mitochondria are dynamic organelles that adapt their morphology and function in response to physiological stimuli, including viral infections. Studying mitochondrial dynamics in mosquito vectors offers valuable insights into cellular homeostasis and mitochondrial function during infection, shedding light on how these arthropods sustain infections throughout their lifespan. Investigating the proteins involved in mitochondrial dynamics is essential for advancing our understanding of vector borne diseases.

## Concluding remarks

This review enhances our understanding of the complex interaction between arboviruses and their arthropod vectors, highlighting metabolic disruptions and adaptations during infection and identifying key biochemical checkpoints critical for arbovirus transmission. Host-derived metabolites such as lipids, sugars, and amino acids, are utilized to facilitate successful arbovirus infection and transmission. Vector tolerance enables pathogen persistence, while resistance involves limiting pathogen load through energetically costly immune responses.

In arthropods, defense mechanisms typically involve increased glycolysis to rapidly produce ATP. Glycolysis also generates significant lactate from pyruvate, suppressing oxidative phosphorylation. Conversely, enhanced OXPHOS and reduced glycolysis result in weaker immune response, increasing vector susceptibility to infection. This balance between metabolic pathways is crucial for determining vector competence. Additionally, VDAC porin has also emerged as a key regulator of viral replication by modulating the apoptosis pathway.

Future studies should further compare mitochondrial OXPHOS and metabolic pathways during arboviral infection in mammalian and arthropod cells ideally in side-by-side analysis, to identify specific alterations that could be targeted to prevent viral persistence in the vector or viral infection in mammalian hosts. Identifying viral mediators of mitochondrial changes that induce resistance or susceptibility in vectors and apoptosis in mammalian cells is a critical area of research with therapeutic potential. Since metabolic manipulation is unlikely to yield an all-or-nothing response, a precise understanding of specific metabolites, metabolic pathways or enzymes involved at each step of the viral cycle is necessary. Such insights inform strategies to disrupt viral infection, replication and spread. Additionally, processes related to cellular signaling pathways, such as ROS production and mitochondrial dynamics, must be characterized in terms of their source, molecular players, intracellular distribution and levels to determine their potential for targeted intervention.

Other metabolic regulators also merit further investigation. For instance, the sirtuin family of proteins has demonstrated significant potential in regulating metabolism under both infectious and non-infectious conditions (Tan et al., 2024; Wang et al., 2018). Similarly, short non-coding RNAs (miRNAs), play a critical role in post-transcriptional gene regulation by binding to target messenger RNAs (mRNAs), inhibiting translation or inducing mRNA degradation (Geiger and Dalgaard, 2017; Shang et al., 2023). Notably, the miRNA let-7 has been identified as a key regulator of metabolic pathways in *B. mori*, targeting pyruvate carboxylase mRNA and emphasizing its role in regulating the anaplerotic TCA cycle (Jitrapakdee et al., 2008; Wang et al., 2020). During viral infection in mammals, these microRNAs regulate a wide range of mRNA targets involved in bioenergetic pathways (Powdrill et al., 2016).

An integrative understanding of the metabolic changes occurring during arboviral infection in arthropods and mammalian cells is essential for developing strategies to manipulate these pathways and modulate viral persistence and infection effectively.
