# Corrigendum: Alteration of mitochondrial function in arthropods during arboviruses infection: a review of the literature

**DOI:** 10.3389/fphys.2025.1578531

**Published:** 2025-03-14

**Authors:** María E. Santana-Román, Santos Ramírez-Carreto, Paola Maycotte, Victoria Pando-Robles

**Affiliations:** ^1^ Centro de Investigaciones Sobre Enfermedades Infecciosas, Instituto Nacional de Salud Pública, Cuernavaca, Morelos, Mexico; ^2^ Centro de Investigación Biomédica de Oriente (CIBIOR), Instituto Mexicano del Seguro Social (IMSS), Puebla, Mexico

**Keywords:** mitochondria, arthropods, infection, arbovirus, metabolite

In the published article, there was an error in the legend for **Figure 2**. The corrected legend appears below.

“Mitochondrial dynamics-related proteins. **(A)** Mitochondria are dynamic organelles that constantly undergo fusion and fission. This figure illustrates the proteins involved in mitochondrial dynamics in mammals while emphasizing the limited data available for arthropods. Arbovirus infection induces cellular stress, disrupting the balance between fusion and fission, which impairs key cellular processes. These processes are represented using a traffic light, with green indicating normal functionality and red signifying inhibition. Created in BioRender.com. **(B–G)** Key proteins involved in mitochondrial fusion and fission are illustrated. Panels **(B–D)** present phylogenetic trees based on amino acid sequences of Mfn-like, Opa1-like, and DRP1-like proteins from relevant arthropods of the genera *Aedes*, *Culex*, and *Anopheles*, as well as *Bombyx mori* were generated using MAFFT software (v7.511). Corresponding proteins from *Drosophila melanogaster* serve as the outgroup. The trees are scaled, with branch lengths representing the evolutionary distances. Numbers represent the sequences used to generate the trees. Groups are color-coded using FigTree (v1.4.4). Panels **(E–G)** present structural alignments for Mfn-like sequences, Opa1 and DRP1, in all cases, the structures of *Drosophila melanogaster* are shown in red, *Aedes aegypti* in black, *Culex pipiens* in yellow, *Anopheles albimanus* in blue and *Bombyx mori* in pink. In all alignments, the GTPase domain is highlighted in dotted circles. Protein sequences were modeled using the ColabFold program (v1.5.5).”

In addition, there were textual errors in the following sections.

A correction has been made to the section **Metabolism in arthropods during arbovirus infection**, Paragraph 3. This sentence previously stated:

“*D. melanogaster* (*D. melanogaster*).”

The corrected sentence appears below:

“*Drosophila melanogaster* (*D. melanogaster*).”

A correction has been made to section **Metabolism in arthropods during arbovirus infection**, Paragraph 7. This sentence previously stated:

“*M. japonicus* (*M. japonicus*).”

The corrected sentence appears below:

“*Litopenaeus vannamei* (*L. vannamei*).”

A correction has been made to section **Energetic substrates and mitochondrial β-oxidation**, Paragraph 2. This sentence previously stated:

“CPT1 inhibition significantly reduces egg viability under stress conditions in Ae. aegypti in Ae. aegypti.”

The corrected sentence appears below:

“CPT1 inhibition significantly reduces egg viability under stress conditions in *Ae. aegypti.*”

A correction has been made to section **Glycolysis pathway and Warburg effect on infection**, Paragraph 3. This sentence previously stated:

“In *Bombyx mori.*”

The corrected sentence appears below:

“In *B. mori.*”

A correction has been made to section **Mitochondrial ROS and protein carriers**, Paragraph 3. This sentence previously stated:

“In *Litopenaeus vannamei* infected with *Vibrio parahaemolyticus.*”

The corrected sentence appears below:

“In *L. vannamei* infected with *Vibrio parahaemolyticus.*”

A correction has been made to section **Mitochondria dynamics**, Paragraph 6. This sentence previously stated:

“including *Ae. aegypti*, *Ae. albopictus*, *A. gambiae*, and *C. pipiens.*”

The corrected sentence appears below:

“including *Ae. aegypti*, *Ae. albopictus*, *A. gambiae*, and *Culex pipiens.*”

A correction has been made to section **Mitochondria dynamics**, Paragraph 8. This sentence previously stated:

“Similarly, RGDV, transmitted by *R. dorsalis*.”

The corrected sentence appears below:

“Similarly, RGDV, transmitted by *Recilia dorsalis*.”

The authors apologize for these errors and state that this does not change the scientific conclusions of the article in any way.

